# Optimization of Green Space Planning to Improve Ecosystem Services Efficiency: The Case of Chongqing Urban Areas

**DOI:** 10.3390/ijerph18168441

**Published:** 2021-08-10

**Authors:** Shuiyu Yan, Jun Tang

**Affiliations:** The School of Architecture and Urban Planning, Chongqing University, Chongqing 400045, China; yanshuiyu@cqu.edu.cn

**Keywords:** landscape indexes, adaptability evaluation, mountainous city, optimization strategies

## Abstract

This paper applied landscape indexes to evaluate the size, form, and structure of green spaces in the mountainous city of Chongqing and found that green spaces benefit from certain advantages in size, but the network suffered from low heterogeneity and limited interconnectivity. To ensure the integrity and continuity of ecological processes and improve the efficiency of ecosystem services (ES), the authors used Geographic Information System (GIS) software to conduct adaptability evaluation and adjacent buffer analysis for the existing green spaces, wetlands, rivers, and other landscapes with relatively high capacity for ES. We designed a comprehensive map of potential areas for UGS expansion by superimposing the maps obtained from adaptability evaluation and buffer analysis. We also proposed some strategies that respect, consider, and evaluate aspects and special features of urban environment to optimize green space planning and improve ES efficiency, such as protection of important areas, development of green corridors, and careful consideration of ecological processes and complex functions in urban areas. Based on these strategies, the paper put forth suggestions for green space planning to improve ES efficiency that can function as foundation for subsequent green space planning.

## 1. Introduction

Urban green spaces (UGS) offer many ecosystem services (ES) that are pivotal in maintaining the quality and stability of the urban environment [1,2], creating leisure and entertainment zones [3,4,5], promoting physical and mental health [6,7], and supporting high-quality urban morphology [8,9]. Ubiquitous urban pollution and natural disasters or extreme phenomena (such as high temperatures, rainstorms, floods), as well as low rankings in livability indexes, relate to the absence or low efficiency of green spaces [10]. Urban planners understood this early on and designed strategies to improve living conditions and environmental stability. For instance, the Open Space Act, promulgated in English law in 1906, incorporated “the open air or open lands without building or with a few buildings, of which the rest parts are functioned by entertainment, recreation, storage and so forth” into the land use management system [11]. Open spaces, such as recreational places, protected areas, and scenic zones with unique characteristics and regulatory effects on the city, are significant considerations during the planning phase in the United States [12]. In European countries, easily accessible urban areas, covered by vegetation and primarily allocated to leisure and entertainment, as well as green spaces with positive impact on the urban environment, are regarded as urban green infrastructure [13]. In China, the planning circle deems important not only natural and semi-natural open spaces (forests, rivers, farmlands, wetlands, and others) that support ecological conservation and protect urban environment, but also semi-artificial and artificial open spaces (urban parks, squares, streets, and others), whose primary function is to meet the demand for recreation activities [14]. All these countries are aware of the significant ecological functions of UGS and consider UGS beneficial for maintaining ecological safety and providing essential ES for human communities.

Many studies have demonstrated that the exertion of ES functions is closely related to the UGS spatial pattern, and the reasonable landscape pattern is crucial to the improvement of ES efficiency. For example, in terms of improving environmental quality, the size, distribution, and form of green spaces can regulate surface temperature [15,16], and strategically located green spaces reduce noise pollution [17,18]. With regard to maintaining biodiversity, the natural landscape pattern (size and connectivity of green patches) and urban development state (urbanization and city compactness) are key factors in shaping a habitat for city birds and supporting biodiversity [19]. Regarding the leisure and entertainment aspects, distance and family income are the main factors affecting the services of parks and recreational facilities, as housing prices within a radius of 1500 m from green spaces are higher than in other areas [20]. In terms of public health, UGS can ameliorate the negative impact of stress on individuals living within a radius of 3 km from green areas [21]. Hence, increasing the number and size of green spaces to make them accessible to more people is beneficial to mental health across local communities [22]. To that end, the optimization of UGS landscape pattern is an effective way to improve the ES efficiency.

Many studies have explored ways to optimize the UGS landscape pattern. Their preferred, frequent recommendation is to increase the number and area size of UGS, because this strategy is easier to implement and operate. For example, Zhang et al. suggested increasing the UGS area as means to improve public health in urban areas [23]; Yao et al. argued that increasing urban green cover would provide significant benefits for rain-water regulation [24]. In fact, the most effective method is to develop green corridors to bolster landscape connectivity [25,26]. In turn, this method requires a UGS landscape structure evaluation, which includes landscape ecological index evaluation [27,28,29], spatial pattern evaluation (e.g., morphological spatial model analysis, accessibility evaluation, and others.) [30,31,32] and ES value evaluation [33,34]. This method focuses on identifying potential green corridors and core functional areas. Based on this, an ecosystem network can be constructed to integrate fragmented habitats and protect biodiversity and regional ecological security [35]. Other studies have also emphasized that the shape of UGS shape is an important factor for ES functions [36,37], and increasing the patch shape index could improve certain ES of UGS [38]. Accordingly, it seems that the optimum UGS landscape pattern should satisfy the following requirements: sufficient quantity and area, reasonable landscape pattern and form, and strong connectivity. This paper addresses a clear gap in our understanding and explores ways to optimize landscape patterns and improve ES efficiency through an assessment of UGS status, adaptability evaluation, and buffer analysis. The paper tackles the following three questions:

- What are the characteristics of the landscape pattern of UGS, and does the current UGS structure need to be optimized?
- Are there enough available spaces to cover future demand for UGS development and promote healthy and sustainable development of ES, if current UGS cannot satisfy future demand for ES?
- How can we identify potential areas for creating more UGS and forming an effective UGS planning to optimize UGS structure and promote ES in Chongqing urban areas?

We examined the mountainous city of Chongqing where four types of UGS can be discerned: regional recreational green spaces (such as forest parks, country parks, or tourist attractions), urban parks, protected areas (natural reserves and wetland protected zones), and other green spaces (such as affiliated green spaces). Firstly, we selected representative landscape indexes to quantitatively evaluate of the UGS size, form, and structure, thereby defining the aspects to be optimized in Chongqing urban areas. Then, we used the GIS software for adaptability evaluation and adjacent buffer analysis of the infrastructure corridors, riverbank, wetland, and other landscape types to determine their potential to accommodate UGS expansion. After superimposing the maps obtained from the above operation, we designed the map of areas with potential for UGS in Chongqing urban areas. Finally, optimization suggestions on green space form, network structure, resilience, and integral function were put forward based on the evaluation results, and we expect them to function as scientific reference and base for optimizing green space planning.

## 2. Materials and Methods

### 2.1. Overview of the Research Area

**Figure 1:**
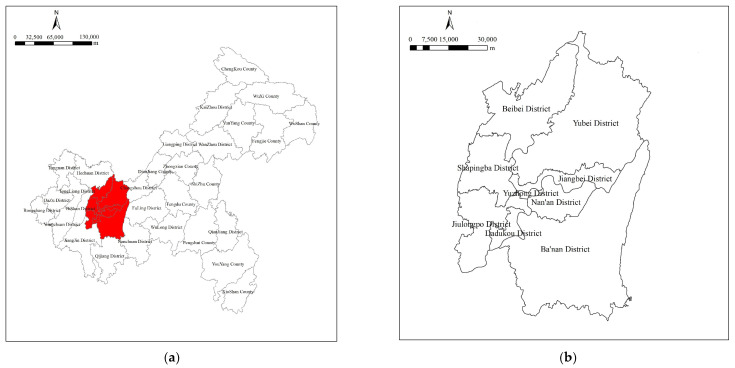
Chongqing urban areas. (**a**) Location of study area: Chongqing urban areas; (**b**) Nine administrative districts of Chongqing urban areas.

Located in the western area of the city, the Chongqing urban areas comprise a total area of approximately 5473 km^2^, a population of 8.65 million, and nine administrative districts: Yuzhong, Jiangbei, Nan’an, Jiulongpo, Shapingba, Dadukou, Beibei, Yubei, and Ba’nan (Figure 1). As a typical mountainous city, the districts of Chongqing are mostly situated on mountainous and hilly lands, as the Jinyun, Zhongliang, Tongluo, Mingyue and other mountains transverse the city from north to south. The Jialing River joins the Yangtze at Chaotian Gate in the Yuzhong district, forming a basic geomorphic entity entailing two rivers and four mountains in Chongqing urban areas. Therein, the current UGS landscape system comprises a variety of types: riverine green spaces along the drainage basins and tributaries of the rivers; green areas around the wetlands and water masses inside the city; mountain ridges and hills. Hence, the urban areas are an important symbiotic environment for fauna and flora, which regulates and improves the quality of life and urban environment, defines the local culture and provides many other services to the city, thus indicating relatively high urban resource capacity.

### 2.2. Research Framework and Data

**Figure 2:**
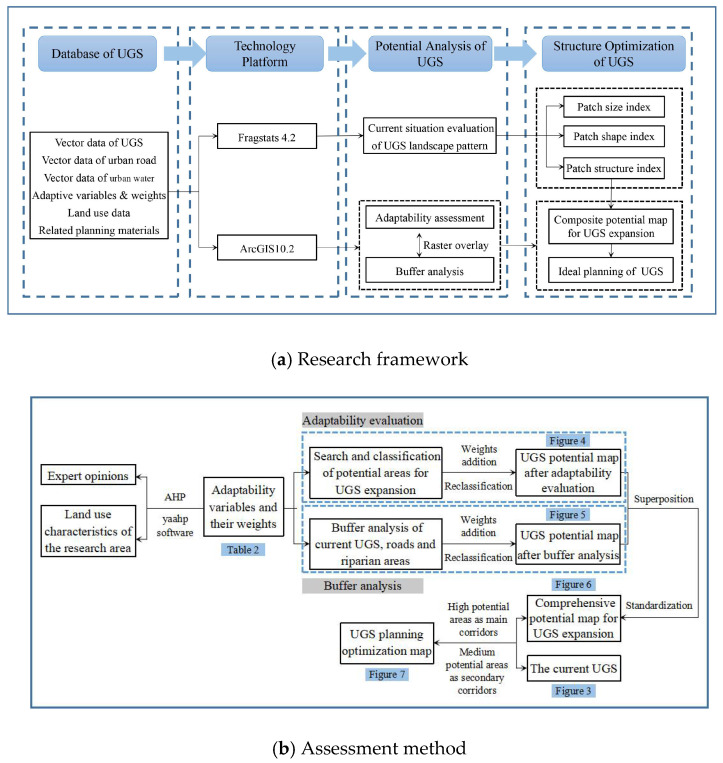
The research technical route. (**a**) The research framework explains the data type, technical platform and overall structure of the paper; (**b**) The evaluation method shows the specific operation methods and steps to determine the potential UGS.

This paper displays the optimization method of UGS planning based on ES efficiency through the evaluation of current UGS status, the optimization of landscape pattern and planning formation. The evaluation mainly focused on the landscape size, form, and structure, while the optimization of landscape pattern was based on the adaptability evaluation of the variables which significantly affect the UGS expansion, and the adjacent buffer analysis regarding the main roads, rivers, and current UGS. We plotted the comprehensive map of potential areas for UGS expansion after weighing the maps obtained from the evaluation. Finally, we designed the optimization planning map of UGS structure in Chongqing urban areas by using areas with great potential to accommodate future UGS development as green corridors to connect current UGS patches and make appropriate adjustments. Detailed steps are shown in the Figure 2a,b.

Vector data and related planning data for UGS, roads, and the river system of Chongqing urban areas in 2019 (including Territorial spatial planning Chongqing municipality (2021–2035), Chongqing overall urban planning (2007–2020) (revised in 2014), Chongqing land use overall planning (2006–2020), and others) were used as data sources and bases of spatial identification for UGS analysis and evaluation (Table 1).

### 2.3. Potential Analysis of UGS

#### 2.3.1. Evaluation of Current UGS Status

The landscape pattern reflects the basic attributes of UGS, and it is closely related to the ecological process and ES functions. The purpose of UGS landscape pattern evaluation is to optimize the current UGS, to rationalize land use, and maximize the services of the landscape ecosystem. We evaluated the UGS landscape pattern from three aspects: size, form and structure. The specific steps are as follows:

Firstly, we reclassified the UGS vector data of Chongqing urban areas for 2019 to design the distribution map of current UGS status. The statistical properties of different patches were taken as the essential data for buffer analysis and adaptability evaluation. Then, we selected 12 representative landscape indexes based existing research [39,40,41] (See Appendix A for the specific calculation formula) and used Fragstats software to evaluate the UGS status.

#### 2.3.2. Adaptability Evaluation and Buffer Analysis

First, we determined the primary variables affecting UGS expansion based on findings of previous research [42], and compared them in pairs using the Analytic Hierarchy Process (AHP). Thus, we defined the adaptability variables and weights that affect UGS expansion (Table 2) after the weighted valuation and priority ordering in line with the degree of relative importance. Next, we searched and classified the potential areas for UGS expansion within the Chongqing urban areas. They were: (a) areas within 100 m of existing UGS patches, (b) wetlands, (c) river banks, (d) open areas or free lands, (e) traffic and infrastructure corridors and (f) demand areas. Afterwards, the potential map after UGS adaptability evaluation was obtained by vectorizing and standardizing major variables.

We built a buffer zone for every variable with the use of GIS software, and the buffer boundaries were determined as follows according to relevant previous research [42]: (1) the existing UGS (200 m for regional recreational green spaces, 50 m for urban parks, 100 m for protected areas, and 50 m for other green spaces); (2) river bank areas (60 m for the Yangtze, 50 m for the Jialing, and 20 m for other tributaries and water areas); (3) wetlands (50 m); and (4) land transportation network (60 m for expressways, 40 m for expressways on arterial roads, and 20 m for arterial roads). We evaluated every potential area of UGS expansion on the basis of adaptability variable weight. The score of each evaluated area comprised the sum of variables’ weight values, if any of the variables shown in Table 2 was relevant to the area; if not, the area was assigned a score of 0. Then, we took the scores of each area as a new field in the attribute table and reclassified them in three tiers: areas of high, medium, and low potential, with a score of 15, 10, and 5, respectively. Similarly, we performed the adjacent buffer analysis for existing UGS, main roads and rivers, and plotted the potential map of adjacent buffer zone after standardization and superposition.

#### 2.3.3. Drawing Map of Potential Areas for UGS Expansion

We drew the comprehensive map of potential areas for UGS expansion in Chongqing urban areas as follows. Firstly, we conducted weighted handling and superposition towards the univariate map produced by the aforementioned buffer analysis and adaptability evaluation. We derived the potential value for each area from the attribute tables and expressed them with figures. Meanwhile, we classified the potential value into three levels (a value of 30 for high-potential areas, 20 for middle-potential areas, and 10 for low-potential areas) and used color gradient to represent comprehensive potential map.

## 3. Results

### 3.1. Evaluation Results of the Current UGS

#### 3.1.1. UGS Size

Ratios of UGS area to total urban area between 2.20% and 13.40% seem to guarantee high-quality ES [33]. The evaluation results in Table 3 (total area of UGS 2090 km^2^, accounting for 38.19% of the total urban area) show that, the UGS in Chongqing urban area has certain advantages in size. As the dominant type in Chongqing urban areas, regional recreational green spaces were few in number but large in surface (accounting for 92.28% of the total UGS area). Green spaces with rich ground vegetation, such as forest parks and country parks, played an important role in carbon sequestration, decrease surface runoff, regulate micro-climate, and support biodiversity. Since large-size urban forests had a great impact on the overall quality of ES (the largest patch index was 27.98%), the managers should clearly delineate protected areas, commit to the protection of forests and improve ES efficiency.

Protected areas, similarly few in number but large in total coverage area, were second only to regional recreational green spaces and some of them have great ecological significance. Therefore, the government should implement reasonable and effective protection strategies to ensure the integrity of the ecological processes and the efficiency of ES. Urban parks and other green spaces featured in relatively large numbers, but covered a small proportion of the area (1.84% and 0.97% of the urban area, and 4.82% and 2.55% of the total UGS area, respectively). We suggest that local authorities and planners increase the area of urban parks and other green spaces by developing new green spaces or changing land types to meet the demand of urban residents for leisure and recreation services.

To summarize, the UGS size of Chongqing urban areas is relatively reasonable, which is indicative of strong natural ecology and resource capabilities and of the ability to provide a variety of compound ecological services for city dwellers. However, the relative scarcity of urban parks and other green spaces (affiliated and protected green spaces) generates urgent demand for better recreation services to residents.

#### 3.1.2. UGS Form

The UGS form in Chongqing urban areas is complex (the Mean Shape Indexes range from 3 to 30) because of the exceptional mountainous terrain; at the same time, this complexity is an advantage in protecting urban biodiversity. However, this extraordinary landscape creates difficulties in some aspects of urban ecology that can affect ES efficiency. For example, mountains can be barriers that hinder the migration of urban species or the pollen dispersal of urban plants; also, they may threaten interior species in cities. Nevertheless, these negative effects can be alleviated by increasing the connectivity between adjacent patches. In addition, the fragmentation of urban parks and other green spaces (patch density is 9.74 and 53.19, respectively) is closely related to infrastructure construction, such as urban road network and municipal pipe gallery. Therefore, UGS shape optimization such as increasing green spaces on both sides of roads and extending green corridors, can improve the ES efficiency of Chongqing urban areas.

#### 3.1.3. UGS Structure

In terms of spatial structure, UGS in Chongqing urban areas is characterized by imbalanced spatial distribution, low landscape heterogeneity and interconnectivity, and the remarkable ecological functions of regional recreational green spaces and protected areas. Landscape diversity and heterogeneity of Chongqing UGS were relatively low (all Shannon Diversity Indexes were lower than 0.5), and so was the landscape connectivity (connectivity indexes were lower than 3). Among the UGS, the spatial distribution of urban parks and other green spaces was more scattered than the two other green spaces that showed more impactful ecological functions and advantages.

To a certain extent, this spatial structure can affect the ecological processes of the urban system and the flow of energy, matter and information, thus threatening the ES efficiency. It is necessary to connect different green spaces with green corridors to form a comprehensive ecological network. Meanwhile, corresponding protection measures, such as strictly controlling land development and transforming areas into new green spaces, should be implemented in areas around green spaces by local authorities and planners.

### 3.2. Landscape Pattern Characteristics of UGS and Optimization Methods

The characteristics of the UGS landscape pattern in Chongqing urban area are as follows:

(1) The UGS size is relatively reasonable; as the dominant landscape type, regional recreational green spaces provide many necessary ES for the city; and scarcity of urban parks and other green spaces (affiliated green spaces) generates pressing demand among residents for better recreation services.
(2) The UGS form is complex, while the fragmentation of urban parks and other green spaces is relatively high.
(3) In terms of UGS spatial structure in Chongqing, it is characterized by unbalanced spatial distribution, low landscape heterogeneity and connectivity, and remarkable ecological functions of regional recreational green spaces and protected areas.

The above results indicate that the UGS size in the study area is generally adequate, but the size of urban parks, and affiliated green spaces need to be expanded. Meanwhile, it is necessary to improve the accessibility and connectivity of UGS with the creation of green corridors. In addition, areas with remarkable ecological functions, such as regional recreational green spaces and protected areas, should be protected. Therefore, we optimized the UGS structure in the following aspects: (1) transforming areas with high potential into new UGS, such as urban parks and affiliated green spaces, (2) selecting the green corridors with high potential for UGS expansion after the adjacent buffer analysis of main roads and rivers, and current UGS to connect existing green patches, (3) building the UGS network system in the optimized map of UGS planning in Chongqing urban areas with the current UGS, the newly expanded green spaces, and the high-potential corridors.

### 3.3. UGS Adaptability Evaluation and Buffer Analysis

#### 3.3.1. Identification of Potential Areas for UGS Expansion and Adaptability Evaluation Results

**Figure 3:**
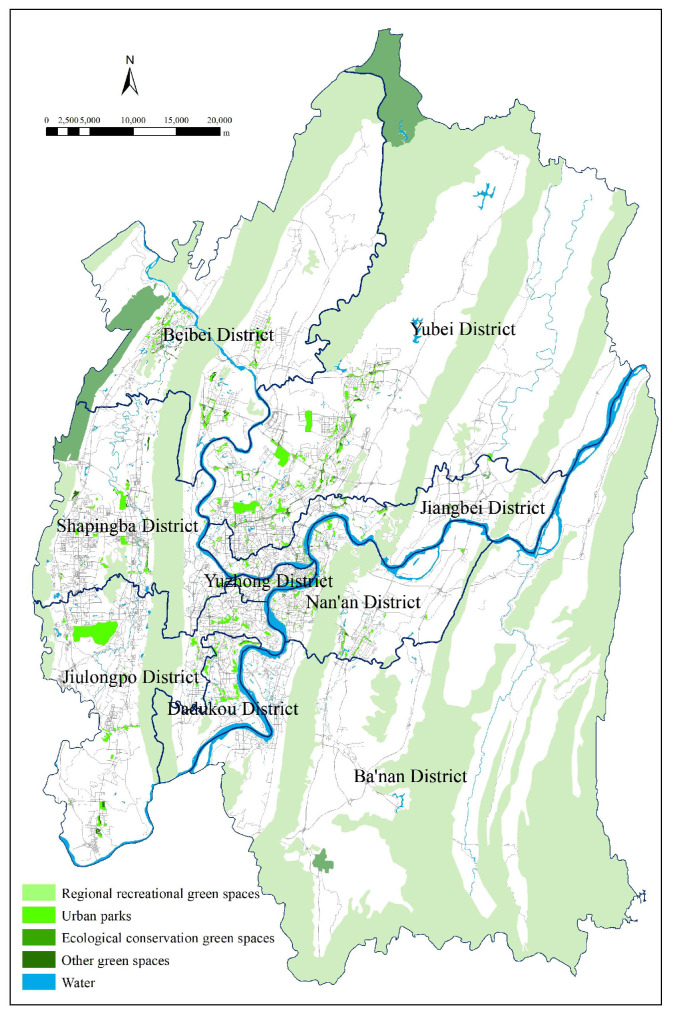
The current urban green spaces.

**Figure 4:**
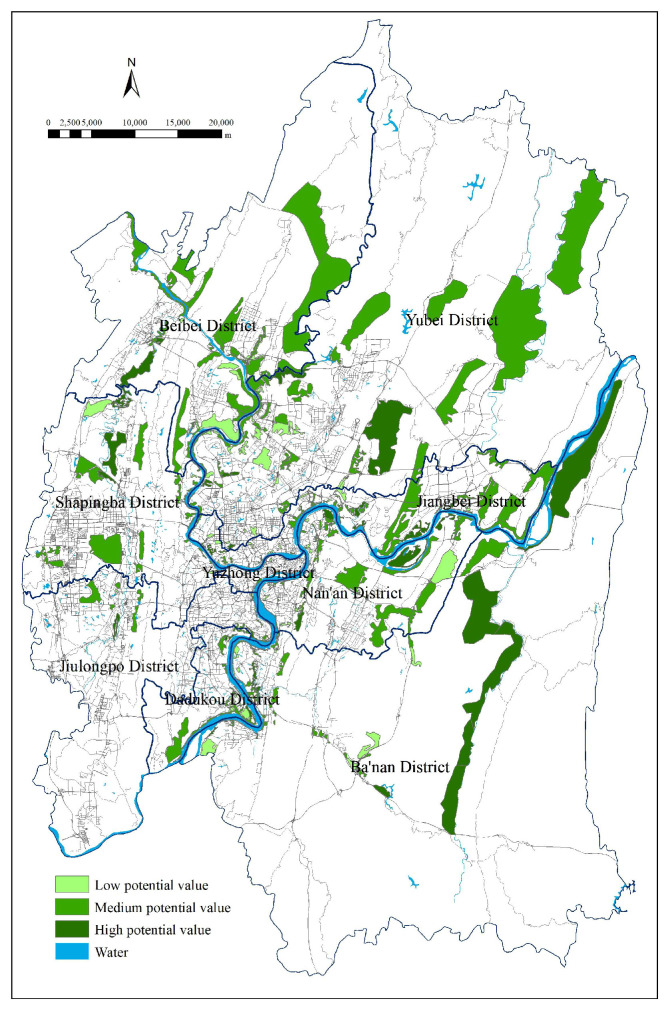
UGS potential map after adaptability evaluation.

As shown in the vector diagram of current UGS status (Figure 3), 937 green space patches of different shapes, sizes, and locations feature within the study area: 30 regional recreational green spaces, 518 urban parks, five protected areas, and 384 other green spaces. W used GIS to identify potential green spaces and analyze their adaptability, and the specific steps were as follows:

(1) there existed 372 potential patchesin total after searching and vectorizing six types of UGS potential expansion areas (i.e., the six adaptive variables in Table 2) one by one,
(2) each patch’s potential to transform into UGS was assessed by adaptive variables and weight tables and the cumulative weight of each potential patch was calculated; the highest value was 0.4639, the lowest 0.0248, and the average value 0.1789,
(3) the potential map after UGS adaptability evaluation (Figure 4) was designed after standardizing the identified potential patches, which were represented with different colours depending on their respective degree of potential (areas of high, medium, and low potential were standardized to 30, 20 and 10, respectively).

#### 3.3.2. Results of the Analysis of UGS Buffer Zones

**Figure 5:**
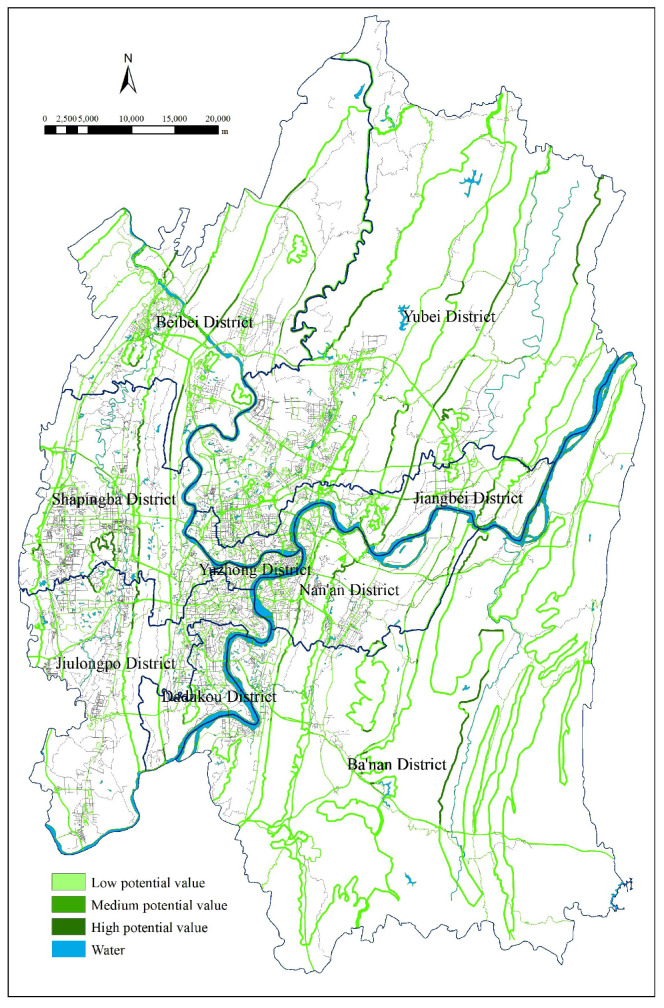
UGS potential map after buffer analysis.

We created corresponding buffer zones and assigned their weights as potential values (0.0393 for current UGS, 0.0741 for roads, 0.0741 for the riparian areas) of current UGS, road network, and water areas using GIS analysis tools. We also used the spatial analysis tools to obtain buffer zones for each variable and their potential value. We superimposed the buffer zones of variables and designed a composite map of UGS adjacent buffer zones (Figure 5) showing a linear green space network traversing the entire study area. In fact, buffer zones along the traffic corridors can effectively reduce pollution, rainwater runoff, and traffic noise, improve environmental conditions, and beautify Chongqing urban areas. In addition, the green corridors formed by buffer zone analysis along the Yangtze and Jialing rivers can improve environmental conditions on both sides of the rivers and the ES efficiency.

#### 3.3.3. Plotting of UGS Comprehensive Potential Map and Effective Planning Map

**Figure 6:**
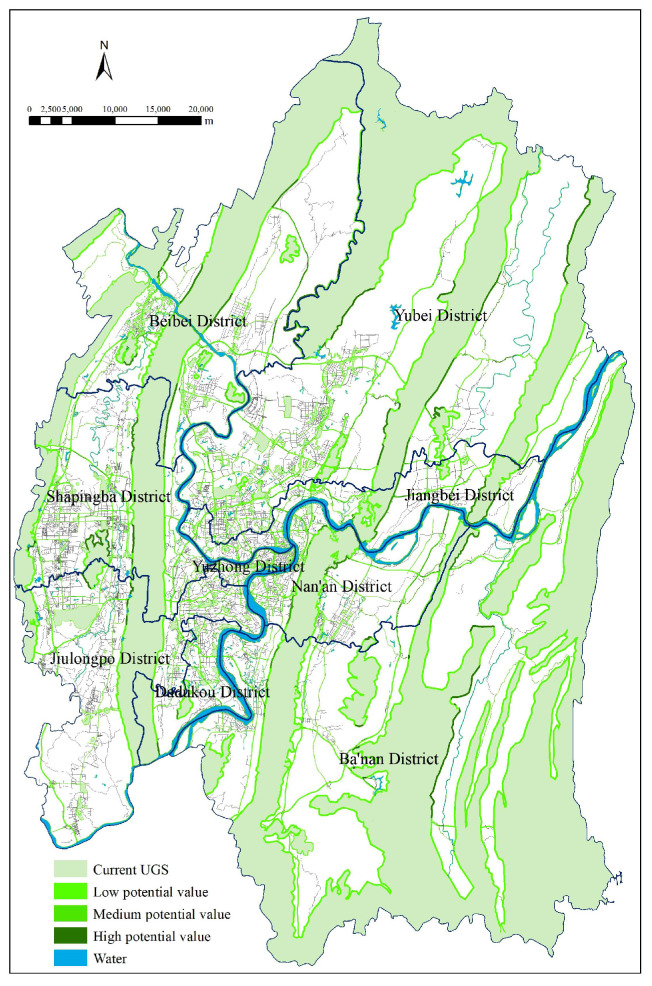
Comprehensive map of potential areas for UGS expansion.

To design the comprehensive map of potential areas for UGS expansion in Chongqing urban areas (Figure 6), we superimposed the two maps (Figure 4 and Figure 5) created as described above and calculated the sum of the superimposed scores for overlapping areas using the single standard score for the non-overlapping ones. The final map displayed a series of potential spaces after increasing interconnectivity in current UGS, which could be part of a green infrastructure system and effectively enhance the service capabilities of the UGS. As shown in Figure 6, regional recreational green spaces, such as urban forest parks and country parks, and the adjacent areas are mostly areas with high potential to accommodate UGS; as such, they are crucial to the improvement of ES efficiency in urban areas, and can be the steppingstones to urban landscape restructuring.

## 4. Discussion

The reasonable pattern of UGS is a physical-space guarantee for sustainable urban development. The quantitative evaluation and structural optimization of UGS facilitate the effective provision and efficiency of urban ES. We answered the three pivotal questions regarding the improvement of the efficiency of urban ES set in the introduction by the evaluation of current UGS status and the identifying areas with potential for UGS development. We also proposed targeted optimization strategies and effective planning schemes to maintain the efficient current form and a high-quality urban environment of Chongqing urban areas.

### 4.1. Three Key Problems Regarding the Improvement of ES Efficiency

The results of the UGS status evaluation, the adaptability evaluation and buffer analysis of related factors in Chongqing urban areas showed that:

- The UGS sizes are basically reasonable, but the structure ought to be optimized. As shown in Table 3, (i) urban forests, wilderness, and other green spaces covered by large areas of green vegetation are enormous in size, thus providing a variety of ES for urban areas, (ii) there is a scarcity in urban parks; as they are closely related to residents’ daily activities, this limits the range of recreation services for urban residents, (iii) the fragmentation of UGS is relatively high; combined with the limited connectivity between and accessibility of green patches, they hinder ecological processes and adversely impact the ES efficiency.
- There are enough areas that can be used to optimize the UGS landscape pattern and improve the efficiency of ES. We provided a total of 372 potential patches to accommodate UGS and displayed the potential areas in the final potential areas map after adaptability evaluation and the assignment of cumulative weights to each potential patch.
- Planning optimization and the identification of potential areas to accommodate UGS were conducted by means of the adaptability evaluation of related factors and the buffer analysis regarding the main roads, rivers, and current UGS (Section 2.3.2).

### 4.2. Structural Characteristics of UGS in Mountainous Areas

The evaluation of the UGS landscape pattern established that the number and size of green spaces in Chongqing urban areas are reasonable, but their form and structure need to be optimized. This conforms to the characteristics of UGS in mountainous areas and the principles of urban development. Similarly to finding of other researchers, a large number of green spaces largely left untouched by human activity are retained in mountainous cities and require minimal or no maintenance to provide a wide range of ES [43]. Green spaces in mountainous cities have unique structure and characteristics owing to their complex geographic and geomorphic conditions and the complex interaction between humans and the environment [30]. Due to the limitations posed by the terrain, mountainous cities are often structured as multicenter clusters. The remaining large green patches (such as regional recreational green spaces and protected areas) between the urban clusters are precious ecological resources and they play an irreplaceable role in protecting biodiversity and improving the urban environment and the quality of residents’ life [31]. As urbanization advances, large green patches may gradually shrink due to the frequent interference of human activities. In their place, smaller, fragmented, scattered, and imbalanced green patches (such as urban parks and other green spaces) might be formed, but they would still be able to provide residents with leisure and recreation, local climate regulation, noise reduction and other services. For this reason, UGS planning methods and rationale that may be effective for other cities should not be introduced to Chongqing without serious consideration of innate environmental differences. Planners ought to respect the internal order of mountainous ecological systems and design and implement holistic strategies suitable for mountainous cities [32]. In what follows, the paper proposes strategies to optimize the planning and development of green spaces for mountainous cities.

### 4.3. UGS Optimization Strategies for Improving the Efficiency of ES

#### 4.3.1. Protecting the Pivotal Functional Spaces in Combination with Environmental Conditions

The mountains and water systems (such as “two rivers and four mountains”) in Chongqing urban areas are large in size, high in ecological value, rich in biodiversity, crucial for the efficiency of ES, but tend to evolve into ecologically sensitive areas particularly vulnerable to pressure from construction activities. The deterioration of vegetation therein will threaten the environmental sustainability and public health, and undermine the living standard and wellbeing of residents. Therefore, urban planners should prioritize these areas, and protect them as the integral part of the UGS framework, whose development and usage should be strictly regulated. In addition, many green spaces in urban areas with small size and complex form (such as urban parks and other green spaces) play an important role in the daily recreation of urban residents, local climate regulation, and noise reduction; consequently, they can be the basic spatial elements of UGS systems to enrich the structure of the ecosystem. To that end, this paper proposes the basic framework of the Chongqing UGS system (Figure 3), with the mountains enveloping the urban area as the matrix and fragmented green spaces as patches.

#### 4.3.2. Optimizing the Green Space Network through Corridor Connection

The primary goal of UGS planning is to fully extend the ecological functions of UGS, so as to protect the urban environment and ecological stability, and emerge as the basic guarantee for the urban sustainable development [31]. The ES of UGS in Chongqing cannot function well without green corridors, while the shapes of most patches are complex, with a small core area and large edge length, which hinders communication between adjacent patches. From the perspective of landscape ecology, all these characteristics facilitate symbiosis within patches and between patches and the external environment. But UGS is also vulnerable to external interference, which undermines its ability to protect interior species and affects biodiversity and stability of the ecosystem. Moreover, green spaces are scattered across Chongqing urban areas loosely and randomly, and it is therefore difficult to create a systematic green space network, while the interconnectivity between patches is compromised. Accordingly, it is even more important to create ecological corridors to connect patches, especially because the overall benefit would be greater than the numerical sum of their respective ecological functions. For this reason, we designed a comprehensive map of potential areas for UGS expansion (Figure 6) obtained by superimposing high-potential linear space with current UGS, which functioned as the basis of UGS planning in Chongqing with reference to the potential map (Figure 4 and Figure 5).

#### 4.3.3. Acknowledging the Role and Importance of Ecological Processes to Optimize the Green Space Planning

**Figure 7:**
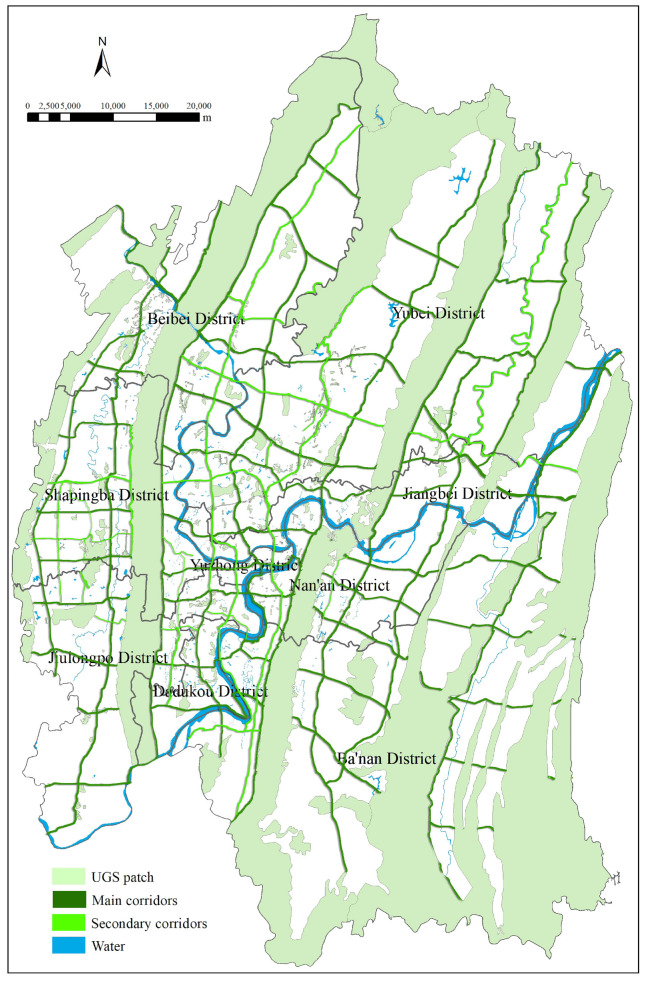
UGS planning optimization of Chongqing urban areas.

A thorough understanding of urban ecological processes is necessary for green space planning in Chongqing urban areas, and so is the optimization of UGS and improvement of ES. The local government should take steps to preserve urban ecosystems by fully considering the spatial characteristics of the mountainous city in Chongqing. We suggest they facilitate the organic connection between urban and green areas by integrating the human-built environment with the natural ecosystem and the composite functions of urban, rural, and natural space in vertical space. This in turn enhances the ecological support and capability of UGS to provide comprehensive services for the city. To that end, we designed a comprehensive potential map of potential areas for UGS expansion and optimized it to construct an “organic, loose, fragmented, and concentrated” UGS model (Figure 7). The purpose was to maintain the integrity and inter-connectivity of UGS and form a healthy and dynamic urban environment.

#### 4.3.4. Appreciating the Composite Functions of Urban Areas to Improve the Resilience of the Urban Ecosystem

Urban planners need to consider both the geographical pattern of UGS and patterns of land use when attempting to improve the ES efficiency in Chongqing urban areas. Firstly, we suggest that planners consolidate the relationship between green spaces and neighboring areas, and enhance current methods in planning methods with a holistic approach to implement the adaptive control measures and strategies. Secondly, relevant departments should divide different control areas of UGS (such as core protected areas, ecological conservation areas, general recreation areas) and refine control measures for pivotal areas based on different characteristics and spatial demands of each district [6]. Finally, urban planners and ecosystem managers should pay attention to the complex interaction between the ecosystem and human society when they protect and restore the ecological environment of Chongqing. In this way, they can coordinate many ES of UGS from an overarching systemic perspective, thereby enhancing the resilience of the whole urban ecosystem and enabling it to cope with unforeseen changes and interventions.

### 4.4. Research Limitations and Future Research

This paper expounded the problems in the green spatial structure of Chongqing urban areas based on the evaluation of UGS patterns and suggested planning schemes to optimize strategies and improve ES efficiency, thus responding to existing problems in urban planning and construction. This endeavor entails a few inevitable limitations. The methods and strategies proposed in this paper revolve around the ES supply efficiency of UGS and may have underplayed the actual demand for ES. The proposed strategy is based on a theoretical exploration of Chongqing current UGS status, and its effects await confirmation from empirical data and practice. The findings of the paper and of similar research in the future can only benefit from an even more systematic examination of UGS planning in mountainous cities. Other researchers may choose to look closer at this aspect to form a set of scientific theories and methods to guide urban planning and construction.

## 5. Conclusions

Quantitative analysis and visual-spatial analysis based on UGS vector data revealed certain advantages in size of the green spaces in Chongqing urban areas, which indicate a relatively strong ecosystem service capability to the city. To make good use of these advantages, the form and structure of UGS must be optimized.

GIS was used to identify potential areas for new UGS in urban areas to facilitate the improvement of landscape pattern and the creation of ecological networks. The purpose was to ensure the integrity and sustainability of ecological processes and constitute the theoretical basis for the structure optimization of UGS and urban ecological planning. On this basis, we suggested the following strategies for green spaces planning optimization: protection of the important green spaces, development of green corridors to connect green patches, consideration of ecological processes to optimize green space planning, and of the complex functions of urban areas to improve the resilience of the urban environment.

This paper showed that GIS technology, especially site selection and spatial identification, can be useful for quantitative analysis and evaluation of UGS. The comprehensive map of potential areas can be the basis for future development of UGS and provides planners and designers with data and theoretical references during the early stages of their analysis, as well as strategic support for planning and operations departments. However, as the fragmentation of UGS and the improvement of ES-efficiency are much more than merely technical issues. Local governments should design and implement effective policies and regulations.

## Figures and Tables

**Table 1:** UGS database of Chongqing urban areas.

Categories	Types	Periods	Applications
Vector data ^1^	Urban green spaces	2019	status evaluation, buffer analysis
	Urban road system	2019	buffer analysis, adaptability assessment
	Urban water system	2019	
Planning data ^2^	Territorial spatial planning Chongqing municipality	2021–2035	UGS amendment, potential areas identification of UGS
	Chongqing overall urban planning	2007–2020	
	Chongqing land use overall planning	2006–2020	
	Chongqing beautiful landscape city planning	2015	
	Regulatory detailed planning of Chongqing urban areas	2006–2020	
Survey data ^3^	Suitability variables and weight values	-	adaptability assessment

^1^ Sourced from: Data Center for Resources and Environmental Sciences, Chinese Academy of Science (RESDC) http://www.resdc.cn (accessed on 23 January 2020). ^2^ Sourced from: Chongqing Planning and Natural Resources Bureau. http://ghzrzyj.cq.gov.cn/zwxx_186/tzgg/202105/t20210527_9335223.html (accessed on 12 June 2020); Ministry of Natural Resources of the People’s Republic of China http://g.mnr.gov.cn/201807/t20180731_2156865.html (accessed on 6 May 2020); Chongqing Planning & Design Institute. ^3^ Source: Professional opinion.

**Table 2:** Adaptability variables and their weights.

Variables	Existing UGS	Wetlands	River Banks	Open Areas or Free Lands	Traffic and Infrastructure Corridors	Demand Areas
Weights	0.0393	0.4669	0.2545	0.1405	0.0741	0.0248

**Table 3:** Evaluation results of current UGS.

Landscape Pattern	Indexes	Regional Recreational Green Spaces	Urban Parks	Ecological Conservation Green Spaces	Other Green Spaces	Summation
Size	Number of Patches (NP)	30	518	5	384	937
	Class Area (CA)/ha	192,888.93	5321.00	10,082.96	721.84	20,9014.73
	Percent of Landscape (PLAND)/%	92.28	2.55	4.82	0.35	100
	Largest Patch Index (LPI)/%	27.98	0.44	2.32	0.02	-
composition	Patch Density (PD)#/km^2^	0.02	9.74	0.05	53.19	-
	Mean Patch Size (MPS)/ha	6884.08	27.52	2496.49	13.14	-
	Mean Shape Index (MSI)	9.65	24.16	3.39	26.59	-
	Mean Patch Fractal Dimension (MPFD)	1.21	1.36	1.13	1.42	-
Structure	Shannon’s Diversity Index (SHDI)	0.11	0.33	0.03	0.37	-
	Dominance Index (LDI)	1.84	1.62	1.92	1.58	-
	Connectance Index (CONNECT)	2.38	1.11	16.67	1.52	-

## Data Availability

The data presented in this study are available on request from the corresponding author. The data are not publicly available due to privacy.

## Appendix A

Landscape index calculation formula

(1) Number of Patches (NP)

NP=n

where, NP≥1, n is the total number of patches of a certain landscape type.

(2) Class Area (CA)

$$CA=\sum_{i=1}^{n}a_{i}$$

where, $a_{i}$ is the area of a certain landscape patch; n is the number of patches.

(3) Percent of Landscape (PLAND)

$$PLAND=\frac{\sum_{j=1}^{n}a_{ij}}{A}$$

where, 0≤PLAND≤100, $a_{ij}$ is the area of the patch; A is the total area of all landscapes.

(4) Largest Patch Index (LPI)

$$LPI=\frac{\max\left(a_{ij}\right)}{A}(100)$$

Here, 0≤ LPI≤100, $a_{ij}$ is the area of the patch; A is the total area of all landscapes.

(5) Patch Density (PD)

$$PD=\frac{n_{i}}{A}$$

where, PD>0, $n_{i}$ is the total area of type i patches; A is the total area of all landscapes.

(6) Mean Patch Size (MPS)

$$MPS=\frac{N}{A}$$

where, *MPS* > 0, N is the total area of the patch; A is the total area of all landscapes.

(7) Mean Shape Index (MSI)

$$MSI=\frac{\sum_{i=1}^{m}\sum_{j=1}^{n}\left(\frac{0.25P_{ij}}{\sqrt{a_{ij}}}\right)}{N}$$

where, MSI>0, $P_{ij}$ is the patch perimeter; $a_{ij}$ is the patch area; N is the total number of patches in the landscape; m is the number of landscape types; n is the number of landscape types of patches.

(8) Mean Patch Fractal Dimension (MPFD)

$$MPFD=\frac{\sum_{i=1}^{m}\sum_{j=1}^{n}\left[\frac{2\ln\left(0.25P_{ij}\right)}{\ln\left(a_{ij}\right)}\right]}{N}$$

where, 1≤ MPFD≤2, $P_{ij}$ is the patch perimeter; $a_{ij}$ is the patch area; N is the total number of patches in the landscape; m is the number of landscape types; n is the number of landscape types of patches.

(9) Shannon’s Diversity Index (SHDI)

$$SHDI=-\sum_{i=1}^{m}\left(p_{i}\ln p_{i}\right)$$

where, $p_{i}$ is the proportion of the area occupied by landscape type i.

(10) Dominance Index (LDI)

$$LDI=H\max+\sum_{i=1}^{m}\left(P_{i}\ln P_{i}\right)$$

where, $H_{max}$ is the maximum diversification index; $p_{i}$ is the proportion of the area occupied by landscape type i.

(11) Connectance Index (CONNECT)

$$CONNECT=\left[\frac{\sum_{i=1}^{m}\sum_{j=i}^{n}C_{ijk}}{\sum_{i=1}^{m}\frac{n_{i}\left(n_{i}-1\right)}{2}}\right]\times 100$$

where, $C_{ijk}$ is the degree of connection between patch j and k in patch type i; $n_{i}$ is the number of patches in patch type i.
